# Supplementary material for: Pregnancy physical activity questionnaire (PPAQ): Translation and cross cultural adaption of an Arabic version
Source: PLoS One. 2020 Mar 30;15(3):e0230420. doi: 10.1371/journal.pone.0230420 (PMC7105115; doi:10.1371/journal.pone.0230420)
Supplement: S1 File — (DOCX) [file pone.0230420.s001.docx]

**S1 File Pregnancy physical activity questionnaire (PPAQ) – Arabic version**

**إستقصاء بالعربية حول النّشاط الجسدي أثناء فترة الحمل**

**خلال الأشهر الثّلاثة (٣) الماضية وخارج نطاق العمل، كم من الوقت كنت تمضين بشكلٍ عام في :**

| **١ . تحضير وجبات الطعام (الطبخ، إعداد المائدة، غسل الآنية)** | **٢ . إلباس أطفالك وغسلهم وإطعامهم وأنت جالسة** | **٣ . إلباس أطفالك وغسلهم وإطعامهم وأنت واقفة** |
| --- | --- | --- |
| - مطلقًا - أقل من _٢_/١ ساعة / اليوم - من _٢_/١ ساعة إلى حوالى ساعة واحدة / اليوم - من ساعة واحدة إلى حوالى ساعتين / اليوم - من ساعتين إلى حوالى ٣ ساعات / اليوم - ٣ ساعات أو أكثر / اليوم | - مطلقًا - أقل من _٢_/١ ساعة / اليوم - من _٢_/١ ساعة إلى حوالى ساعة واحدة / اليوم - من ساعة واحدة إلى حوالى ساعتين / اليوم - من ساعتين إلى حوالى ٣ ساعات / اليوم - ٣ ساعات أو أكثر / اليوم | - مطلقًا - أقل من _٢_/١ ساعة / اليوم - من _٢_/١ ساعة إلى حوالى ساعة واحدة / اليوم - من ساعة واحدة إلى حوالى ساعتين / اليوم - من ساعتين إلى حوالى ٣ ساعات / اليوم - ٣ ساعات أو أكثر / اليوم |
| **٤ . اللّعب مع الأطفال وأنت جالسة أو واقفة** | **٥. اللّعب مع الأطفال وأنت تمشين أو تركضين** | **٦ . حمل أولادك (بين ذراعيك، بواسطة حمّالة الأطفال، على ظهرك، إلخ)** |
| - مطلقًا - أقل من _٢_/١ ساعة / اليوم - من _٢_/١ ساعة إلى حوالى ساعة واحدة / اليوم - من ساعة واحدة إلى حوالى ساعتين / اليوم - من ساعتين إلى حوالى ٣ ساعات / اليوم - ٣ ساعات أو أكثر / اليوم | - مطلقًا - أقل من _٢_/١ ساعة / اليوم - من _٢_/١ ساعة إلى حوالى ساعة واحدة / اليوم - من ساعة واحدة إلى حوالى ساعتين / اليوم - من ساعتين إلى حوالى ٣ ساعات / اليوم - ٣ ساعات أو أكثر / اليوم | - مطلقًا - أقل من _٢_/١ ساعة / اليوم - من _٢_/١ ساعة إلى حوالى ساعة واحدة / اليوم - من ساعة واحدة إلى حوالى ساعتين / اليوم - من ساعتين إلى حوالى ٣ ساعات / اليوم - ٣ ساعات أو أكثر / اليوم |

**خلال الأشهر الثّلاثة (٣) الماضية وخارج نطاق العمل، كم من الوقت كنت تمضين بشكلٍ عام في :**

| **٧ . الإهتمام بشخصٍ مسنٍّ** | **٨. الجلوس لاستخدام الحاسوب أو للكتابة خارج دوام العمل** | **٩ . مشاهدة التلفاز، شريط فيديو أو د.ف.د. (أسطوانة رقميّة)** |
| --- | --- | --- |
| - مطلقًا - أقل من _٢_/١ ساعة / اليوم - من _٢_/١ ساعة إلى حوالى ساعة واحدة / اليوم - من ساعة واحدة إلى حوالى ساعتين / اليوم - من ساعتين إلى حوالى ٣ ساعات / اليوم - ٣ ساعات أو أكثر / اليوم | - مطلقًا - أقل من _٢_/١ ساعة / اليوم - من _٢_/١ ساعة إلى حوالى ساعة واحدة / اليوم - من ساعة واحدة إلى حوالى ساعتين / اليوم - من ساعتين إلى حوالى ٣ ساعات / اليوم - ٣ ساعات أو أكثر / اليوم | - مطلقًا - أقل من _٢_/١ ساعة / اليوم - من _٢_/١ ساعة إلى حوالى ساعة واحدة / اليوم - من ساعة واحدة إلى حوالى ساعتين / اليوم - من ساعتين إلى حوالى ٣ ساعات / اليوم - ٣ ساعات أو أكثر / اليوم |

| **١٠ . الجلوس للقراءة، للتّحدّث إلى أحدهم أو للإتّصال بأحدهم خارج دوام العمل** | **١١ . اللّعب مع الحيوانات الأليفة** | **١٢ . القيام بالأعمال المنزليّة اليومية (ترتيب السرير، الغسيل، الكيّ، ترتيب الأغراض)** |
| --- | --- | --- |
| - مطلقًا - أقل من _٢_/١ ساعة / اليوم - من _٢_/١ ساعة إلى حوالى ساعة واحدة / اليوم - من ساعة واحدة إلى حوالى ساعتين / اليوم - من ساعتين إلى حوالى ٣ ساعات / اليوم - ٣ ساعات أو أكثر / اليوم | - مطلقًا - أقل من _٢_/١ ساعة / اليوم - من _٢_/١ ساعة إلى حوالى ساعة واحدة / اليوم - من ساعة واحدة إلى حوالى ساعتين / اليوم - من ساعتين إلى حوالى ٣ ساعات / اليوم - ٣ ساعات أو أكثر / اليوم | - مطلقًا - أقل من _٢_/١ ساعة / اليوم - من _٢_/١ ساعة إلى حوالى ساعة واحدة / اليوم - من ساعة واحدة إلى حوالى ساعتين / اليوم - من ساعتين إلى حوالى ٣ ساعات / اليوم - ٣ ساعات أو أكثر / اليوم |
| **١٣ . التّبضّع (شراء الطعام، الملابس وحاجيّات أخرى)** | **١٤ . تنظيف المنزل (إستخدام المكنسة، غسل النّوافذ)** |  |
| - مطلقًا - أقل من _٢_/١ ساعة / اليوم - من _٢_/١ ساعة إلى حوالى ساعة واحدة / اليوم - من ساعة واحدة إلى حوالى ساعتين / اليوم - من ساعتين إلى حوالى ٣ ساعات / اليوم - ٣ ساعات أو أكثر / اليوم | - مطلقًا - أقل من _٢_/١ ساعة / الأسبوع - من _٢_/١ ساعة إلى حوالى ساعة واحدة / الأسبوع - من ساعة واحدة إلى حوال ساعتين / الأسبوع - من ساعتين إلى حوالى ٣ ساعات / الأسبوع - ٣ ساعات أو أكثر / الأسبوع |  |

- ***التنقّل من مكانٍ لآخر...***

**خلال الأشهر الثّلاثة (٣) الماضية، كم من الوقت كنت تمضين بشكلٍ عام في :**

| **١٥ . المشي البطيء للوصول إلى مكانٍ معيّن (مثال: ركوب الباص/السيارة، الذّهاب إلى العمل، القيام بزيارة) وليس بداعي المتعة أو لممارسة الرياضة** | **١٦ . المشي السريع للوصول إلى مكانٍ معيّن (مثال: ركوب الباص/السيارة، الذّهاب إلى العمل أو إلى المدرسة) وليس بداعي المتعة أو لممارسة الرياضة** | **١٧ . قيادة السيّارة أو ركوبها أو ركوب الباص** |
| --- | --- | --- |
| - مطلقًا - أقل من _٢_/١ ساعة / اليوم - من _٢_/١ ساعة إلى حوالى ساعة واحدة / اليوم - من ساعة واحدة إلى حوالى ساعتين / اليوم - من ساعتين إلى حوالى ٣ ساعات / اليوم - ٣ ساعات أو أكثر / اليوم | - مطلقًا - أقل من _٢_/١ ساعة / اليوم - من _٢_/١ ساعة إلى حوالى ساعة واحدة / اليوم - من ساعة واحدة إلى حوالى ساعتين / اليوم - من ساعتين إلى حوالى ٣ ساعات / اليوم - ٣ ساعات أو أكثر / اليوم | - مطلقًا - أقل من _٢_/١ ساعة / اليوم - من _٢_/١ ساعة إلى حوالى ساعة واحدة / اليوم - من ساعة واحدة إلى حوالى ساعتين / اليوم - من ساعتين إلى حوالى ٣ ساعات / اليوم - ٣ ساعات أو أكثر / اليوم |

- ***في سبيل المتعة أو ممارسة الرياضة...***

**خلال الأشهر الثّلاثة (٣) الماضية، كم من الوقت كنت تمضين بشكلٍ عام في :**

| **١٨ . المشي البطيء في سبيل المتعة أو لممارسة الرياضة** | **١٩ . المشي السريع في سبيل المتعة أو لممارسة الرياضة** | **٢٠ . المشي السريع صعودًا في سبيل المتعة أو لممارسة الرياضة** |
| --- | --- | --- |
| - مطلقًا - أقل من _٢_/١ ساعة / الأسبوع - من _٢_/١ ساعة إلى حوالى ساعة واحدة / / الأسبوع - من ساعة واحدة إلى حوالى ساعتين / / الأسبوع - من ساعتين إلى حوالى ٣ ساعات / الأسبوع - ٣ ساعات أو أكثر / الأسبوع | - مطلقًا - أقل من _٢_/١ ساعة / الأسبوع - من _٢_/١ ساعة إلى حوالى ساعة واحدة / / الأسبوع - من ساعة واحدة إلى حوالى ساعتين / / الأسبوع - من ساعتين إلى حوالى ٣ ساعات / الأسبوع - ٣ ساعات أو أكثر / الأسبوع | - مطلقًا - أقل من _٢_/١ ساعة / الأسبوع - من _٢_/١ ساعة إلى حوالى ساعة واحدة / الأسبوع - من ساعة واحدة إلى حوالى ساعتين / / الأسبوع - من ساعتين إلى حوالى ٣ ساعات / الأسبوع - ٣ ساعات أو أكثر / الأسبوع |
| **٢١ . الركض** | **٢٢ . متابعة صفوف تمرينيّة للحوامل** | **٢٣ . السباحة** |
| - مطلقًا - أقل من _٢_/١ ساعة / الأسبوع - من _٢_/١ ساعة إلى حوالى ساعة واحدة / الأسبوع - من ساعة واحدة إلى حوالى ساعتين / / الأسبوع - من ساعتين إلى حوالى ٣ ساعات / الأسبوع - ٣ ساعات أو أكثر / الأسبوع | - مطلقًا - أقل من _٢_/١ ساعة / الأسبوع - من _٢_/١ ساعة إلى حوالى ساعة واحدة / الأسبوع - من ساعة واحدة إلى حوالى ساعتين / / الأسبوع - من ساعتين إلى حوالى ٣ ساعات / الأسبوع - ٣ ساعات أو أكثر / الأسبوع | - مطلقًا - أقل من _٢_/١ ساعة / الأسبوع - من _٢_/١ ساعة إلى حوالى ساعة واحدة / الأسبوع - من ساعة واحدة إلى حوالى ساعتين / / الأسبوع - من ساعتين إلى حوالى ٣ ساعات / الأسبوع - ٣ ساعات أو أكثر/ الأسبوع |
|  | **هل تقومين بأمورٍ أخرى بداعي المتعة أو لممارسة الرياضة ؟ يُرجى تسميتها.** | |
| **٢٤ . الرّقص** | **٢٥ . ـــــــــــــــــــــــــــــــــــــــــــــــــــــ**  **إسم النشاط** | **٢٦ . ـــــــــــــــــــــــــــــــــــــــــــــــــــــ**  **إسم النشاط** |
| - مطلقًا - أقل من _٢_/١ ساعة / الأسبوع - من _٢_/١ ساعة إلى حوالى ساعة واحدة / الأسبوع - من ساعة واحدة إلى حوالى ساعتين / / الأسبوع - من ساعتين إلى حوالى ٣ ساعات / الأسبوع   ٣ ساعات أو أكثر / الأسبوع | - مطلقًا - أقل من _٢_/١ ساعة / الأسبوع - من _٢_/١ ساعة إلى حوالى ساعة واحدة / الأسبوع - من ساعة واحدة إلى حوالى ساعتين / / الأسبوع - من ساعتين إلى حوالى ٣ ساعات / الأسبوع - ٣ ساعات أو أكثر / الأسبوع | - مطلقًا - أقل من _٢_/١ ساعة / الأسبوع - من _٢_/١ ساعة إلى حوالى ساعة واحدة / الأسبوع - من ساعة واحدة إلى حوالى ساعتين / الأسبوع - من ساعتين إلى حوالى ٣ ساعات / الأسبوع - ٣ ساعات أو أكثر / الأسبوع |

- ***أثناء العمل ...***

**رجاءً إملئي القسم الآتي إذا كنت تعملين لقاء أجرٍ أو تقومين بعملٍ تطوّعيٍّ أو لا تزالين طالبة. أمّا إذا كنت ربّة منزلٍ، في إجازةٍ الأمومة في المنزل، عاطلة عن العمل أو غير مؤهّلة للعمل فأنت لا تحتاجين لتملئي هذا القسم الأخير.**

**خلال الأشهر الثّلاثة (٣) الماضية، كم من الوقت كنت تمضين بشكلٍ عام في :**

| **٢٧ . الجلوس أثناء العمل أو في الصّف** | **٢٨ . الوقوف أو المشي البطيء أثناء العمل وأنت تحملين أغراضًا أثقل من غالون حليب (سعة ٤ ليتر)** | **٢٩ . الوقوف أو المشي البطيء أثناء العمل من دون أن تحملي أيّ شيء** |
| --- | --- | --- |
| - مطلقًا - أقل من _٢_/١ ساعة / اليوم - من _٢_/١ ساعة إلى حوالى ساعة واحدة / اليوم - من ساعة واحدة إلى حوالى ساعتين / اليوم - من ساعتين إلى حوالى ٣ ساعات / اليوم - ٣ ساعات أو أكثر / اليوم | - مطلقًا - أقل من _٢_/١ ساعة / اليوم - من _٢_/١ ساعة إلى حوالى ساعة واحدة / اليوم - من ساعة واحدة إلى حوالى ساعتين / اليوم - من ساعتين إلى حوالى ٣ ساعات / اليوم - ٣ ساعات أو أكثر / اليوم | - مطلقًا - أقل من _٢_/١ ساعة / اليوم - من _٢_/١ ساعة إلى حوالى ساعة واحدة / اليوم - من ساعة واحدة إلى حوالى ساعتين / اليوم - من ساعتين إلى حوالى ٣ ساعات / اليوم - ٣ ساعات أو أكثر / اليوم |
| **٣٠ . المشي السريع أثناء العمل وأنت تحملين أغراضًا أثقل من غالون حليب (سعة ٤ ليتر)** | **٣١ . المشي السريع أثناء العمل من دون أن تحملي أيّ شيء** |  |
| - مطلقًا - أقل من _٢_/١ ساعة / اليوم - من _٢_/١ ساعة إلى حوالى ساعة واحدة / اليوم - من ساعة واحدة إلى حوالى ساعتين / اليوم - من ساعتين إلى حوالى ٣ ساعات / اليوم - ٣ ساعات أو أكثر / اليوم | - مطلقًا - أقل من _٢_/١ ساعة / اليوم - من _٢_/١ ساعة إلى حوالى ساعة واحدة / اليوم - من ساعة واحدة إلى حوالى ساعتين / اليوم - من ساعتين إلى حوالى ٣ ساعات / اليوم - ٣ ساعات أو أكثر / اليوم |  |

**PREGNANCY PHYSICAL ACTIVITY QUESTIONNAIRE**

During the last three months, when you are NOT at work, in general, how much time do you usually spend :

1. **Preparing meals (cook, set table, wash dishes)**

- None
- Less than ½ hour per day
- ½ to almost 1 hour per day
- 1 to almost 2 hours per day
- 2 to almost 3 hours per day
- 3 or more hours per day

2. **Dressing, bathing, feeding children while you are sitting**

- None
- Less than ½ hour per day
- ½ to almost 1 hour per day
- 1 to almost 2 hours per day
- 2 to almost 3 hours per day
- 3 or more hours per day

3. **Dressing, bathing, feeding children while you are standing**

- None
- Less than ½ hour per day
- ½ to almost 1 hour per day
- 1 to almost 2 hours per day
- 2 to almost 3 hours per day
- 3 or more hours per day

4. **Playing with children while you are sitting or standing**

- None
- Less than ½ hour per day
- ½ to almost 1 hour per day
- 1 to almost 2 hours per day
- 2 to almost 3 hours per day
- 3 or more hours per day

5. **Playing with children while you are walking or running**

- None
- Less than ½ hour per day
- ½ to almost 1 hour per day
- 1 to almost 2 hours per day
- 2 to almost 3 hours per day
- 3 or more hours per day

6. **Carrying children (on your lap, back, …)**

- None
- Less than ½ hour per day
- ½ to almost 1 hour per day
- 1 to almost 2 hours per day
- 2 to almost 3 hours per day
- 3 or more hours per day

7. **Taking care of an older adult**

- None
- Less than ½ hour per day
- ½ to almost 1 hour per day
- 1 to almost 2 hours per day
- 2 to almost 3 hours per day
- 3 or more hours per day

8. **Sitting and using a computer or writing, while you are not at work**

- None
- Less than ½ hour per day
- ½ to almost 1 hour per day
- 1 to almost 2 hours per day
- 2 to almost 3 hours per day
- 3 or more hours per day

9. **Watching TV or a video or DVD**

- None
- Less than ½ hour per day
- ½ to almost 1 hour per day
- 1 to almost 2 hours per day
- 2 to almost 3 hours per day
- 3 or more hours per day

10. **Sitting and reading, talking, or on the phone, while not at work**

- None
- Less than ½ hour per day
- ½ to almost 1 hour per day
- 1 to almost 2 hours per day
- 2 to almost 3 hours per day
- 3 or more hours per day

11. **Playing with pets**

- None
- Less than ½ hour per day
- ½ to almost 1 hour per day
- 1 to almost 2 hours per day
- 2 to almost 3 hours per day
- 3 or more hours per day

12. **Light cleaning (arranging beds, laundry, iron, put things away)**

- None
- Less than ½ hour per day
- ½ to almost 1 hour per day
- 1 to almost 2 hours per day
- 2 to almost 3 hours per day
- 3 or more hours per day

13. **Shopping (for food, clothes, or other items)**

- None
- Less than ½ hour per day
- ½ to almost 1 hour per day
- 1 to almost 2 hours per day
- 2 to almost 3 hours per day
- 3 or more hours per day

14. **Heavier cleaning (vacuum, mop, sweep, wash windows)**

- None
- Less than ½ hour per week
- ½ to almost 1 hour per week
- 1 to almost 2 hours per week
- 2 to almost 3 hours per week
- 3 or more hours per week

**Going places**

During the last three months, how much time do you usually spend:

15. **Walking slowly to go places (such as to the bus, work, visiting) Not for fun or exercise**

- None
- Less than ½ hour per day
- ½ to almost 1 hour per day
- 1 to almost 2 hours per day
- 2 to almost 3 hours per day
- 3 or more hours per day

16. **Walking quickly to go places (such as to the bus, work, or school) Not for fun or exercise**

- None
- Less than ½ hour per day
- ½ to almost 1 hour per day
- 1 to almost 2 hours per day
- 2 to almost 3 hours per day
- 3 or more hours per day

17. **Driving or riding in a car or bus**

- None
- Less than ½ hour per day
- ½ to almost 1 hour per day
- 1 to almost 2 hours per day
- 2 to almost 3 hours per day
- 3 or more hours per day

**For fun or exercise**

**During the last three months, how much time do you usually spend:**

18. **Walking slowly for fun or exercise**

- None
- Less than ½ hour per week
- ½ to almost 1 hour per week
- 1 to almost 2 hours per week
- 2 to almost 3 hours per week
- 3 or more hours per week

19. **Walking more quickly for fun or exercise**

- None
- Less than ½ hour per week
- ½ to almost 1 hour per week
- 1 to almost 2 hours per week
- 2 to almost 3 hours per week
- 3 or more hours per week

20. **Walking quickly up hills for fun or exercise**

- None
- Less than ½ hour per week
- ½ to almost 1 hour per week
- 1 to almost 2 hours per week
- 2 to almost 3 hours per week
- 3 or more hours per week

21. **Jogging**

- None
- Less than ½ hour per week
- ½ to almost 1 hour per week
- 1 to almost 2 hours per week
- 2 to almost 3 hours per week
- 3 or more hours per week

22. **Prenatal exercise class**

- None
- Less than ½ hour per week
- ½ to almost 1 hour per week
- 1 to almost 2 hours per week
- 2 to almost 3 hours per week
- 3 or more hours per week

23. **Swimming**

- None
- Less than ½ hour per week
- ½ to almost 1 hour per week
- 1 to almost 2 hours per week
- 2 to almost 3 hours per week
- 3 or more hours per week

24. **Dancing**

- None
- Less than ½ hour per week
- ½ to almost 1 hour per week
- 1 to almost 2 hours per week
- 2 to almost 3 hours per week
- 3 or more hours per week

**Doing other things for fun or exercise? Please tell us what they are.**

25.­­­­­­­ ­_______________________________

Name of activity

- None
- Less than ½ hour per week
- ½ to almost 1 hour per week
- 1 to almost 2 hours per week
- 2 to almost 3 hours per week
- 3 or more hours per week

26. ________________________________

Name of activity

- None
- Less than ½ hour per week
- ½ to almost 1 hour per week
- 1 to almost 2 hours per week
- 2 to almost 3 hours per week
- 3 or more hours per week

**Please fill out the next section if you work for wages, as a volunteer, or if you are a student. If you are a homemaker, out of work, or unable to work, you do not need to complete this last section.**

**At Work…**

**During the last three months, how much time do you usually spend:**

**27. Sitting at working or in class**

- None
- Less than ½ hour per day
- ½ to almost 1 hour per day
- 1 to almost 2 hours per day
- 2 to almost 3 hours per day
- 3 or more hours per day

28. **Standing or slowly walking at work while carrying things (heavier than a 1 L. milk jug)**

- None
- Less than ½ hour per day
- ½ to almost 1 hour per day
- 1 to almost 2 hours per day
- 2 to almost 3 hours per day
- 3 or more hours per day

29. **Standing or slowly walking at work without carrying anything**

- None
- Less than ½ hour per day
- ½ to almost 1 hour per day
- 1 to almost 2 hours per day
- 2 to almost 3 hours per day
- 3 or more hours per day

30. **Walking quickly at work while carrying things (heavier than a 1 L milk jug)**

- None
- Less than ½ hour per day
- ½ to almost 1 hour per day
- 1 to almost 2 hours per day
- 2 to almost 3 hours per day
- 3 or more hours per day

31. **Walking quickly at work without carrying anything**

- None
- Less than ½ hour per day
- ½ to almost 1 hour per day
- 1 to almost 2 hours per day
- 2 to almost 3 hours per day
- 3 or more hours per day

**Thank you**
