# Supplementary material for: Pregnancy physical activity questionnaire (PPAQ): Translation and cross cultural adaption of an Arabic version
Source: PLoS One. 2020 Mar 30;15(3):e0230420. doi: 10.1371/journal.pone.0230420 (PMC7105115; doi:10.1371/journal.pone.0230420)
Supplement: Attachment — Submitted filename: Rebutal letter PPAQ.docx [file pone.0230420.s003.docx]

The authors present an article about an interesting topic: adaptation of The Pregnancy Physical Activity Questionnaire (PPAQ) to Arabian conditions. However, it is necessary to justify the choice of instrument. Maybe it would be more informative the use of a specific questionnaire for pregnant women than a general questionnaire, e.g. Pregnancy Sympton Inventory – PSI (Foxcroft KF, Callaway LK, Byrne NM, Webster J. Development and validation of a pregnancy symptoms inventory. BMC Pregnancy Childbirth. 2013;13:3. Published 2013 Jan 16. doi:10.1186/1471-2393-13-3). The PSI provides a comprehensive inventory of pregnancy related symptoms, with a mechanism for assessing their effect on function. It was robustly developed, with good test re-test reliability, face validity, comprehension and readability. This provides a validated tool for assessing the impact of interventions in pregnancy.

Thank you for sharing with us this research.

It is with a great interest that we read the article concerning the development of a pregnancy symptoms inventory, however we could not have use it in our study, hence the main aim of our research was to assess the physical activity level and not the pregnancy related symptoms such tiredness, nausea or other pregnancy related problems in our population.

The major mistypes have been detected:

1. The structure of the Abstract does not comply with PLoS One guidelines.

We arranged the structure of the abstract as mentioned in Plos One guidelines.

The aim is not clearly defined.

The aim of the research is divided into primary and secondary objectives and mentioned at the end of the introduction. Thank you.

In addition to adapting PPAQ to Arabic conditions, the purpose of the work is - as I understand it - to study the relationship of physical activity (PA) of pregnant women with their age, crowding index and pregestational body mass index.

We evaluated the physical activity level of our participants and studied the maternal factors that might influence it.

Abbreviations should not be used in the Abstract.

Done. They were removed. Thank you.

The authors write that they analyze the socioeconomic status - which is not consistent with the paper.

A section was added in Methods concerning the crowding index.

By the way, this is a big limitation of this study.

We admit that socioeconomic status influence the physical activity level of adults, however the added-value of our tool, the PPAQ, is that it takes into consideration different types of activities, such as occupational type or leisure type or sport-oriented type. Hence this will permit to differentiate between active and sedentary females.

Physical activity in pregnancy may depend on the socio-economic status and support of a partner, friends or family.

We admit that socioeconomic status and the encouragement of family or friends motivate people to become more active, however women of low socioeconomic status will rarely engage in sports, because in Middle-East, societal pressure and religious beliefs limit the possibilities of outdoor activities. Hence, the majority of women should adhere to a private club and pay a membership (monthly or annually) to be able to practice sports.

2. The Introduction does not present the background of the paper. There is no information on the state of knowledge regarding PA in pregnant women. There are many publications on this subject, e.g .:

- Krzepota J., Sadowska D., Biernat E. Relationship between physical activity and quality of life in pregnant woman in the second and third trimester. International Journal of Environmental Research and Public Health 2018: 15: 2745.
- Gjestland, K., Bø, K., Owe, K. M., & Eberhard-Gran, M. (2012). Do pregnant women follow exercise guidelines? Prevalence data among 3482 women, and prediction of low-back pain, pelvic girdle pain and depression. Br J Sports Med, bjsports-2012.
- Fell, D. B., Joseph, K. S., Armson, B. A., & Dodds, L. (2009). The impact of pregnancy on physical activity level. Maternal and Child Health Journal, 13(5), 597–603.
- Evenson, K. R., Savitz, A., & Huston, S. L. (2004). Leisure‐time physical activity among pregnant women in the US. Paediatric and Perinatal Epidemiology, 18(6), 400–407.

New references were added in the introduction. Thank you for sharing with us those studies.

Line 48-60 - this fragment should be moved to the Method chapter

Done. Thank you for your remark.

3. **Statistical Analysis**

Line 153 – it is necessary to make the following sentence more specific: 1 MET is the metabolic equivalent of the energy expended at rest and equals 3.5 ml O_2_/kg/min.

Done.

4. **Results**

Line 184 - missing ”p”, as in line 222

Line 190 –it is necessary to mark significant differences* in tab. 3

Line 199 - full stop is not placed at the end of legend

In Tab. 2-6 it is necessary to standardize the p value record in the legend. In tab. 2 and 3 there is: *p value less than 0.05 considered as significant*. In tab. 4, 5, 6 - ** P < 0.05*

Line 205 – standardize the lower case p (e.g. line 182), the capital P (e.g. line 205)

Line 224-226 – this fragment should be moved to Discussion

All corrections concerning this section were taken into consideration. Thank you.

5. Apart from the Introduction, the **Discussion** is the weakest part of the paper. The Authors refer to data from 35 years ago (line 236).

This reference was replaced by a recent publication. Thank you.

Line 237-240 – The sentence *Although the ACOG recommends pregnant females to maintain a moderately steady intensity level of exercise, yet a low percentage of pregnant women exercise at recommended levels, which is significantly lower when compared to the general population (“ACOG Committee Opinion No. 650,” 2015)* is not clear. Which women? Lebanese?

The sentence was reformulated. We were referring to pregnant women in general, not Lebanese. Thank you.

Line 249 – where does this conclusion come from? *As expected, PA increased during the second trimester in our sample*. The results indicate that it only refers to household/caregiving and light intensity PA.

The sentence was reformulated. Thank you.

Line 258-260 – instead of 52% it should be 51,4%

Done.

Line 266 - *Watching television was the sedentary behavior that the majority of women spent, similar in the study of Santos PC et al (Santos et al., 2016) and Chasan-Taber et al (Chasan-Taber et al., 2004a).* How do we know it? How does it relate to the phrase in line 174? -

*18% had a sedentary lifestyle*

The items 8,9, and 10 of the PPAQ Arabic version relate to the time spent for reading, using the computer and watching the television. These reflected in the sedentary behavior of the participants.

Line 267 i 268 - the first letter of the name is not necessary

Done. Thank you.

Line 268 – The following sentence raises controversy: *Chandonnet N. et al. applied the French version of PPAQ among pregnant obese women who were predominantly active in household and caregiving activities, as in our sample specially among housewives, or those with no higher academic education (Chandonnet et al., 2012).* The quoted study can only be compared to obese participants. Have the authors examined the professional status? Where does this conclusion come from? Besides, the quote (Chandonnet et al., 2012) should appear after Chandonnet N. et al. – and not at the end of the sentence, because this part of the sentence regards the study of the Authors.

The sentence was reformulated and all the remarks were corrected. Thank you.

Line 273-276 –In the sentence *Results derived from this study showed positive correlations regarding maternal age and total PA ( P= 0.034), however no associations were seen between PPAQ scores and pregestational BMI, gestational age, worry or insomnia (Mourady et al., 2017)* it is not clear which results refer to the study by Mourady et al., 2017, and which refer to the Authors’ study. Moreover, worry and insomnia were not tested.

The sentence was reformulated. The study of Mourady et al. used our translated PPAQ among another sample of participants, with other variables of interest such as worry and insomnia. Thank you.
