# Supplementary material for: Pregnancy physical activity questionnaire (PPAQ): Translation and cross cultural adaption of an Arabic version
Source: PLoS One. 2020 Mar 30;15(3):e0230420. doi: 10.1371/journal.pone.0230420 (PMC7105115; doi:10.1371/journal.pone.0230420)
Supplement: Attachment — Submitted filename: Response to reviewers.docx [file pone.0230420.s004.docx]

Rebuttal Letter

Dear editor and reviewers,

On behalf of all the authors, we highly appreciate your valuable comments and recommendations, that were fully taken into consideration in the final revised submitted manuscript. Hope it will meet your requirements.

PONE-D-19-25377R1
Pregnancy physical activity questionnaire (PPAQ): translation and cross cultural adaption of an Arabic version.
PLOS ONE

Dear Dr Papazian,

Thank you for submitting your manuscript to PLOS ONE. After careful consideration, we feel that it has merit but does not fully meet PLOS ONE’s publication criteria as it currently stands. Therefore, we invite you to submit a revised version of the manuscript that addresses the points raised during the review process.

Abstract

- Line 7-9: Please also indicate one of the objectives of the study was to measure the physical activity of pregnant women using the adapted PPAQ. Done.
- The methods sub-section is very superficial and does not clearly describe the four steps followed in the adaptation process. An explanation was added.
- The results sub-section looks deficient because it does not report on the cross-cultural adaptations made to the PPAQ tool, which is one of the objectives of the study.  Please revise accordingly. Done.
- “with a significant p-value of 0.031 and 0.001 respectively” which specific groups are these p-values referring to? Household? Caregiving? occupational activities? An explanation was added.
- “Our results reported that 51% of women engage in light intensity physical activity. Please also report % who had high intensity exercise and sedentary life style. Done.

Background

- Line 38-9: Can you put a citation for the claim “In addition, due to religious sensitivities, exercising in public areas or local gyms are forbidden for women in some Middle-Eastern countries”. A citation was added.

Methods

- Line 76-76: please provide citation for the sentence “Our research team followed the guidelines depicted by Guillemin et al., which were composed of 4 phases, to guarantee the reliability of the translated tool”. Done.
- In the methods section please clearly describe how the 179 student participants were selected for the study and how this sample size was reached at. An explanation was provided.

Results

- Line 177-8: “The mean BMI calculated based on their pregestational weight was 22.94± 3.60 kg/m” how did you manage to determine the pre-pregnancy weight? Please describe in the methods section. An explanation was provided.
- Line 180-182 and table-1: it is not clear what you mean by “self-reported nutritional status” and “self-reported physical well-being”. Please also clearly describe in the methods section how these variables were measured. An explanation was provided.
- Line 192: “Significant correlations……” please change to “significant association”. Done

Discussion

- Line 250-1: “This ensured the homogeneity of our sample.”??? Not clear please rephrase it again?
- Line 315 “The strength of our study is the homogeneity of the sample”. Homogeneity on the basis of what?

Corrections were done and the term “homogeneity” was removed from the manuscript. Thank you.

We would appreciate receiving your revised manuscript by Feb 28 2020 11:59PM. When you are ready to submit your revision, log on to https://www.editorialmanager.com/pone/ and select the 'Submissions Needing Revision' folder to locate your manuscript file.

If you would like to make changes to your financial disclosure, please include your updated statement in your cover letter.

To enhance the reproducibility of your results, we recommend that if applicable you deposit your laboratory protocols in protocols.io, where a protocol can be assigned its own identifier (DOI) such that it can be cited independently in the future. For instructions see: <http://journals.plos.org/plosone/s/submission-guidelines#loc-laboratory-protocols>

Please include the following items when submitting your revised manuscript:

- A rebuttal letter that responds to each point raised by the academic editor and reviewer(s). This letter should be uploaded as separate file and labeled 'Response to Reviewers'.
- A marked-up copy of your manuscript that highlights changes made to the original version. This file should be uploaded as separate file and labeled 'Revised Manuscript with Track Changes'.
- An unmarked version of your revised paper without tracked changes. This file should be uploaded as separate file and labeled 'Manuscript'.

Please note while forming your response, if your article is accepted, you may have the opportunity to make the peer review history publicly available. The record will include editor decision letters (with reviews) and your responses to reviewer comments. If eligible, we will contact you to opt in or out.

We look forward to receiving your revised manuscript.

Kind regards,

Samson Gebremedhin, PhD
Academic Editor
PLOS ONE


Additional Editor Comments (if provided):

Please also make sure that the following comments, which had been forward by one of the reviewers during the first-round review, had been accommodated.

The authors present a work of interest and the translation of a widely used tool during pregnancy, that is useful to use in other countries and languages. Also, it gives a general insight of the PA levels of Lebanese pregnant women for the first time, being a topic of interest. However, the presentation of the results and discussion needs review, as well as some part of the statistical analysis. The full text need a deep revision of the English writing, as well as the construction of several sentences.
The manuscript was corrected by a colleague, whose native language was English. A regression analysis was done to improve the statistical outcomes and the results. The discussion section was reviewed as well.

Abstract
Line 5. Please change PA level for “levels” throughout the text, as it refers to different intensities.
Done.

Line 7-9. Please rephrase the sentence or watch the punctuation, as it is difficult to follow.

Done.

Line 13. Please change females for women throughout the text.
Done.

Line 19-20. Compare to what? To first trimester? Or to other types of activities? Please specify in abstract. Also, the second trimester does not have the highest results for occupational activities, if you see the respective table.
Corrections were done. Thank you.

Introducition
Page 9 line 41. Change benefice for benefits.
Done.
Line 41-44. Could you cite any of those questionnaires that are too long or subjective?

Done.

How is PPAQ not subjective, as it is a self-administred questionnaire. Please rewrite if necessary.
Corrections were done. This was added in the limitation section too.

Line 51. Add to the sentence that is the amount of time spent in the specific activity.

This is the sentence in line 51: “A valid PA tool in Arabic is necessary to help researchers assess, monitor and evaluate PA in North African and Middle Eastern pregnant females”.

We added as suggested: A valid PA tool in Arabic is necessary to help researchers assess, monitor and evaluate PA as well as the amount of time spent in each specific activity, in North African and Middle Eastern pregnant females.

Is this what you proposed?

Line 51. Start the sentence with Six instead of 6.

There is no 6 in the line 51.

Methods
Line 100. Don’t know if I understand well. The 45 pregnant females were all part of the university staff? If not, please, rephrase.

The participants of the pilot phase of the study were from our university community (lecturers, employees, students) and the affiliated hospital staff (nurses, employees, dietitians, doctors…).

Line 109. Mentioned where? In the introduction you say PA is important together with nutrition, but no mentioning of using another questionnaire.

Data in relation with the nutritional status and physical activity will be presented in another article.

Furthermore, even if this particular study does not need that specific information as you used another complimentary questionnaire to assess that, maybe another study will use the full information, and therefore, the final Arabic version should take into account all the questions.

Done. We can add to the translated PPAQ to be shared in the future with other researchers those questions related to the date of recruitment, last menstrual period, and the predicted date of delivery.

Line 118. There are no other activities more usual in the Middle East that could be changed for those activities with a similar MET expenditure? This way the totals could be comparable with studies from other countries using the PPAQ.

In Middle Eastern and Arabic countries, religious constraints, lack of green spaces, and societal traditions don’t offer new choices or behaviors of physical activity.

Line 120. Please change “our” environment for “Middle Eastern” or “Arabic” countries.

Done

Line 127. Please cite the sutyd of there is a clinical trial or pprevious work published.

After reviewing publications on Pubmed and to our knowledge, our study is the first to evaluate physical activity level among women in Arab countries and the Middle-East. Our research team conducted in parallel, another study, with different objectives, among a different sample of women, and used our translated version of the PPAQ. We provided in our manuscript, some details related to this study and cited the proper reference.

Line 129. Singleton baby or singleton pregnancy?

Singleton pregnancy. It was corrected in the text. Thank you.

Line 134. Please refer to self-administer the newly translated PPAQ

It was mentioned in line 144 as follow:

“The last part of the questionnaire, concerned the newly translated PPAQ and was self-filled by the participants themselves”


Line 154. Please specify that the final number was 2 less in your study and you took that into account in the corresponding kind of activity/intensity totals.

We did not understand the comment. Line 154 is a subtitle. What do you suggest? If you can explain further please.

Statistical Analyses. Please indicate the corresponding table of results to each of the statistical analyses described.

The authors don’t specify any confounders for the ANOVA analyses comparing the PPAQ results by groups. However, when comparing, for example the PA levels by high education of low education (table 2) they don’t take into account how many women there are of each trimester of gestation, in each group. Even more, given the information that the PA levels differ significantly during the different trimesters, they may consider it as confounder. The same would apply for other tables and confounders.

In order to control confounding factors, univariates analyses followed by multivariate analyses were performed to assess the association between each explanatory variable and the PPAQ. A new table was added at the end of the result section, as follows.

**Table 7. Multiple regression analysis of explanatory factors associated with PPAC in women**

|  | Unstandardized Coefficients | | Standardized Coefficients | t | Sig. | 95.0% Confidence Interval for B | |
| --- | --- | --- | --- | --- | --- | --- | --- |
|  | B | Std. Error | Beta |  |  | Lower Bound | Upper Bound |
| (Constant) | 176.566 | 71.302 |  | 2.476 | .014 | 35.831 | 317.300 |
| Maternal age | 1.654 | 1.281 | .096 | 1.291 | .198 | -.875 | 4.183 |
| Education | 20.560 | 15.499 | .099 | 1.327 | .186 | -10.031 | 51.150 |
| Nutritional status [Ref: very bad/bad] | -70.038 | 25.462 | -.202 | -2.751 | .007 | -120.294 | -19.782 |
| Physical status [Ref: very bad / Bad] | 41.538 | 17.988 | .171 | 2.309 | .022 | 6.034 | 77.043 |

Results
162 add “of pregnancy” at the end of the sentence.

Done.

Line 204-205. Please keep the hypothesis and subjective approach for the discussion section with the proper citation.
These are lines 204-205:

“… sedentary lifestyle with a significant p value of 0.001 compared with females in their first or second trimester. In addition, household chores were significantly higher in women, in their second..”

in these lines, there is no hypothesis or subjective approach, they are results of the study that we present.

Tables. In all titles of the tables, please add “sedentary time” to the “Type and intensity of PA”. Also, please add units of measurement.

Done. Thank you.

Line 222. Add “respectively” after the P values.

Done.

Lines 224-228. Please put this information into the discussion section.

Line 224-228 are in fact the table 4 and not a text. So we did not understand the comment.

Discussion.
Line 251. The authors state “PA increased during the second trimester in our sample. Indeed, this period is considered to be the most comfortable period during gestation, where women often feel themselves more energetic and eager to be physically active”, while in the table, the total score for PPAQ is higher in the third trimester than the second, as well as in some particular intensities and activities. Also, please use references when explaining your results.

All this section was reviewed and corrected. New references were added.

Line 253-256. Please rephrase into shorter sentences (there is twice the word “since”)

The sentence was reformulated and corrected accordingly. Thank you.

277. Highlights.

Done

286-287. Final part of the sentence is not clear.

Corrections were done. Thank you.

294. How is the sample homogenous if there are women of all gestational ages and from different studies and economic backgrounds?
The sentence was reformulated and the word “homogenous” was deleted. Thank you.
Conclusions.
Line 303. Please rephrase the last sentence.
Done.
Line 310. Women
Done.

[Note: HTML markup is below. Please do not edit.]

Reviewers' comments:


[NOTE: If reviewer comments were submitted as an attachment file, they will be attached to this email and accessible via the submission site. Please log into your account, locate the manuscript record, and check for the action link "View Attachments". If this link does not appear, there are no attachment files to be viewed.]


While revising your submission, please upload your figure files to the Preflight Analysis and Conversion Engine (PACE) digital diagnostic tool, <https://pacev2.apexcovantage.com/>. PACE helps ensure that figures meet PLOS requirements. To use PACE, you must first register as a user. Registration is free. Then, login and navigate to the UPLOAD tab, where you will find detailed instructions on how to use the tool. If you encounter any issues or have any questions when using PACE, please email us at [figures@plos.org](mailto:figures@plos.org). Please note that Supporting Information files do not need this step.
